# Efficacy of Electronic Reminders in Increasing the Enhanced Recovery After Surgery Protocol Use During Major Breast Surgery: Prospective Cohort Study

**DOI:** 10.2196/44139

**Published:** 2023-11-03

**Authors:** Sumeet Gopwani, Ehab Bahrun, Tanvee Singh, Daniel Popovsky, Joseph Cramer, Xue Geng

**Affiliations:** 1 Department of Anesthesiology MedStar Georgetown University Hospital Washington, DC United States; 2 Georgetown University School of Medicine Washington, DC United States; 3 Department of Anesthesiology, Perioperative and Pain Medicine Icahn School of Medicine at Mount Sinai New York, NY United States; 4 Department of Biostatistics, Bioinformatics & Biomathematics Georgetown University Washington, DC United States

**Keywords:** ERAS protocol, electronic notification system, clinical decision support system, postoperative outcomes, breast surgery, surgery, surgical, postoperative, decision support, notification, recovery, anesthesia, cohort study, patient outcome, enhanced recovery, patient education, surgical stress

## Abstract

**Background:**

Enhanced recovery after surgery (ERAS) protocols are patient-centered, evidence-based guidelines for peri-, intra-, and postoperative management of surgical candidates that aim to decrease operative complications and facilitate recovery after surgery. Anesthesia providers can use these protocols to guide decision-making and standardize aspects of their anesthetic plan in the operating room.

**Objective:**

Research across multiple disciplines has demonstrated that clinical decision support systems have the potential to improve protocol adherence by reminding providers about departmental policies and protocols via notifications. There remains a gap in the literature about whether clinical decision support systems can improve patient outcomes by improving anesthesia providers’ adherence to protocols. Our hypothesis is that the implementation of an electronic notification system to anesthesia providers the day prior to scheduled breast surgeries will increase the use of the already existing but underused ERAS protocols.

**Methods:**

This was a single-center prospective cohort study conducted between October 2017 and August 2018 at an urban academic medical center. After obtaining approval from the institutional review board, anesthesia providers assigned to major breast surgery cases were identified. Patient data were collected pre- and postimplementation of an electronic notification system that sent the anesthesia providers an email reminder of the ERAS breast protocol the night before scheduled surgeries. Each patient’s record was then reviewed to assess the frequency of adherence to the various ERAS protocol elements.

**Results:**

Implementation of an electronic notification significantly improved overall protocol adherence and several preoperative markers of ERAS protocol adherence. Protocol adherence increased from 16% (n=14) to 44% (n=44; *P*<.001), preoperative administration of oral gabapentin (600 mg) increased from 13% (n=11) to 43% (n=43; *P*<.001), and oral celebrex (400 mg) use increased from 16% (n=14) to 35% (n=35; *P*=.006). There were no statistically significant differences in the use of scopolamine transdermal patch (*P*=.05), ketamine (*P*=.35), and oral acetaminophen (*P*=.31) between the groups. Secondary outcomes such as intraoperative and postoperative morphine equivalent administered, postanesthesia care unit length of stay, postoperative pain scores, and incidence of postoperative nausea and vomiting did not show statistical significance.

**Conclusions:**

This study examines whether sending automated notifications to anesthesia providers increases the use of ERAS protocols in a single academic medical center. Our analysis exhibited statistically significant increases in overall protocol adherence but failed to show significant differences in secondary outcome measures. Despite the lack of a statistically significant difference in secondary postoperative outcomes, our analysis contributes to the limited literature on the relationship between using push notifications and clinical decision support in guiding perioperative decision-making. A variety of techniques can be implemented, including technological solutions such as automated notifications to providers, to improve awareness and adherence to ERAS protocols.

## Introduction

Standardizing practices in a variety of industries has proven to be effective in the productivity and quality of work. With respect to perioperative care, a major development of standardized evidence-based practice is implementing clinical guidelines and protocols, such as enhanced recovery after surgery (ERAS) protocols [1]. ERAS protocols are multidisciplinary evidence-based guidelines for the care of surgical patients. The aim is to minimize the stress of surgery and support patients to recover as soon as possible. ERAS protocols have increasingly been shown to improve postoperative outcomes for patients in a variety of surgical procedures [2,3]. In particular, it has been found to reduce hospital stay lengths and improve patient outcomes in a cost-effective manner [2]. Protocol use in the health care setting is increasingly seen as a driver of quality and safety via a standardization of practice. However, adherence to ERAS protocols by providers has been a challenge. One reason could be that ERAS protocols are considered complex and resource-demanding [2]. Introducing protocols usually requires a major shift in clinical practice and many health care providers have difficulties making these changes [2].

Fortunately, studies have demonstrated that clinical decision support systems (CDSS) for anesthesia have the potential to improve protocol adherence by reminding providers about departmental policies and protocols [1-5]. A CDSS system consists of three modules: (1) data acquisition through the electronic health record or anesthesia information management system, (2) processing of data through rules modulation, and (3) notification to the health care provider [6]. Several examples of CDSS systems can be noted within anesthesiology and perioperative management, such as devices that alert clinicians to abnormalities in blood pressure readings, or systems that warn of excessive anesthetic being administered [6]. It is clear that CDSS systems offer many applications in knowledge management and passive provider education [6]. CDSS have seen a rapid evolution since their first use in the 1980s. These systems are now commonly administered through electronic medical records or other computerized clinical workflows [6]. Several industries across the services and technology sector have implemented the use of push notifications as a primary method to improve communications to targeted users. Notification systems have also been implemented within the health care setting as a means of CDSS [7]. Here, we examine the use of these push notifications to increase ERAS protocol adherence by providers caring for patients undergoing major breast surgery. Our hypothesis is that the implementation of an electronic notification system to anesthesia providers the day prior to scheduled breast surgeries will increase the use of the ERAS protocols. We hypothesize that this notification system will draw providers’ attention to the protocol enhancing use and improving patient outcomes.

## Methods

### Recruitment

This was a single-center prospective cohort study conducted between October 2017 and August 2018 at MedStar Georgetown University Hospital, a tertiary academic medical center in Washington DC. Participants met the inclusion criteria if they were attending anesthesiologists, anesthesiology residents, certified registered nurse anesthetists, and student registered nurse anesthetists in the department assigned to major breast surgery cases. Attending anesthesiologists involved in writing the ERAS protocol, and anesthesia providers in this study were excluded from participation. For the purposes of this study, mastectomies and breast reconstruction such as deep inferior epigastric perforator flaps were the only 2 surgical procedures that were considered major breast surgeries. Other procedures performed by breast surgeons were not included in this analysis.

The current focus of ERAS at our institution is preoperative and intraoperative interventions such as premedication and anesthetic technique, that is, avoiding narcotics. For the purpose of this study, an increase in guideline adherence was defined as a 20% increase in the use of scopolamine, gabapentin, and celecoxib. The specific protocol can be seen in Multimedia Appendix 1. Previous studies of ERAS protocols have used different rates to exhibit adherence. A 20% increase in protocol use was set due to previous research using this standard to define adherence [8]. The preintervention database included 100 patients who underwent major breast surgery from October 2017 to January 2018. Similarly, the postintervention database included 100 patients who underwent major breast surgery from February to August 2018. Based on this study’s power analysis, 83 patients in each category were needed to detect the predefined outcome with statistical significance. The sample size needed to achieve adequate power, in this case, set at 80%, was done using a web-based clinical calculator called ClinCalc. This calculator uses study group design, primary end point, and statistical parameters such as anticipated incidence and type 1 and 2 error rate in order to calculate the size needed to achieve a set power value.

Using an institutional electronic medical record, a database was created, which identified patients who were undergoing major breast surgery. Patient data were anonymous and deidentified. For the postintervention group, practitioners assigned to operating rooms with these surgeries were also identified the day prior to surgery, and the following notification was sent to the respective anesthesia provider via email:

We have identified that you will be in breast surgery tomorrow, where the attached Early Recovery after Surgery (ERAS) protocol may or may not be applicable. Please consider whether this protocol is appropriate for each of your patients individually. The attending anesthesiologist will make the final determination of appropriate care for each patient.

The purpose of electronic notifications was to remind providers that ERAS protocols are in place rather than have them use traditional human and system factors. Electronic notifications were emailed to providers in an automated fashion at 7 PM every night prior to surgery using a CDSS built by the study team. The CDSS included data acquisition, data processing, and provider notification modules. Anesthesiology staff assignments and operating room schedules are uploaded as PDF documents onto a WordPress-based departmental website. Data acquisition was done with a custom-designed AppleScript to extract the schedules from the website each evening and process them through optical character recognition software (Adobe Acrobat Pro DC). In the Apple Xcode (Apple, Cupertino) development environment, using the C++ programming language, an algorithm was written to process the schedules, parsing the text with a delimiter function. This allowed the identification of providers assigned to major breast surgery. A notification script (AppleScript) was implemented to push emails to providers with instructions as well as the ERAS protocol for breast surgery. Anesthesia providers used the ERAS protocol contained within the email and did not use any premade order sets.

Data were collected from the patient’s electronic medical chart in both the pre- and postintervention groups including the date and type of surgery as well as the type of anesthesia provided. For the purposes of this study, the focus was on the medications that were administered preoperatively, intraoperatively, and postoperatively. This included the use of gabapentin, celecoxib, acetaminophen, ketamine, scopolamine, fentanyl, and ondansetron.

The primary outcome of this study was to measure the adherence to the ERAS protocol, which is determined by preoperative and intraoperative use of medications. Preoperative medications used were as follows: oral gabapentin 600 mg, oral celebrex 400 mg, oral acetaminophen 1000 mg for AM admissions, and transdermal scopolamine patch. Intraoperative medications used were as follows: midazolam PRN, propofol gtt, ketamine 20 mg IV ± gtt at 0.2 mg/kg/h, decadron 8 mg IV at induction, ondansetron 4 mg IV prior to extubation, and acetaminophen 1000 mg IV if not given preoperatively. Secondary outcomes included intraoperative and postoperative narcotics administered, postanesthesia care unit (PACU) length of stay (LOS), first pain score, highest pain scores postoperatively, and incidence of postoperative nausea and vomiting (PONV). Narcotics used in this study included: fentanyl, morphine, dilaudid, and oxycodone, which were considered to be morphine equivalents. In the ERAS protocol sent to providers, there was a distinct pathway that detailed the following stages: preoperative clinic, preoperative holding, intraoperative, and postoperative stages. Each patient care stage was clearly labeled with the appropriate pharmacologic interventions required, specifically, what dosage of the drugs previously mentioned was appropriate and whether the drug was administered intravenously or orally.

### Ethical Considerations

Approval was obtained from the MedStar Health Research Institute institutional review board (STUDY #2017-0725), and informed consent was obtained from all patients. Secondary analysis of data was allowed per MedStar Health institutional review board protocol after obtaining initial primary consent. Retrospective data were analyzed in accordance with MedStar Health’s nonhuman subjects research policies.

### Statistical Analysis

All statistical analyses were performed using the statistical software RStudio (version 1.4.1106; Posit). For this study, we accepted a *P* value less than .05 for statistical significance. The initial data included 200 participants, with 100 in the prenotification group and 100 in the postnotification group. Data characteristics were summarized by frequency and percentage for categorical variables and mean and SD or median and IQR for continuous variables based on the normality of the data. Shapiro-Wilk test was used to check the normality of the continuous variables. The following variables were used in analyses between groups: patient age, weight, American Society of Anesthesiology (ASA) score, PACU LOS, first pain score, highest pain score, intraoperative morphine equivalent, and postoperative morphine equivalent. Student 2-sided *t* test or Wilcoxon rank-sum test was used to check whether there was a significant association between continuous variables and categorical variables based on the normality of the continuous variables. Chi-square test was used to determine whether there was a significant association between 2 categorical variables, and Fisher exact test was used instead of chi-square test if a cell count was less than 5.

## Results

### Participants’ Demographic Information

In total, 12 patients in the preintervention group who had minor breast surgery were excluded from all analyses. Additionally, 3 patients from the preintervention group and 1 patient from the postintervention group were also excluded from all analyses due to missing data. Demographic variables of the patients undergoing breast surgery are shown in Table 1. The variable patient age was normally distributed, whereas the following 7 continuous variables were not distributed normally: weight, ASA score, PACU LOS, first pain score, highest pain score, intraoperative morphine equivalent, and postoperative morphine equivalent. A total of 176 patient cases were included in this study; 85 cases where anesthesia providers were in the pre-electronic notification group and 99 cases where the anesthesia providers were in the postelectronic notification group. Of the 85 participants providing anesthetic care in the pre-electronic notification group, the mean age of patients undergoing breast surgery was 50.81 (SD 12.65) years, the median weight was 70.8 (IQR 61.50-84.00) kg, and the median ASA score was 2.00 (IQR 2.00-3.00). Of the 99 participants providing anesthetic care in the postelectronic notification group, the mean age of patients undergoing breast surgery was 50.74 (SD 14.30) years, the median weight was 76 (IQR 61.35-87.60) kg, and the median ASA score was 2.00 (IQR 2.00-3.00). There were no statistically significant differences in mean age, median weight, and median ASA score (*P*>.05) between the groups.

**Table 1:** Baseline characteristics of patients included in the study (N=176).

Characteristic	Pre-electronic notification	Postelectronic notification	*P* value
Age (years), mean (SD)	50.81 (12.65)	50.74 (14.30)	.97
Weight (kg), median (IQR)	70.8 (61.50-84.00)	76.00 (61.35-87.60)	.25
ASA^a^ score, median (IQR)	2.00 (2.00-3.00)	2.00 (2.00-3.00)	.82

^a^ASA: American Society of Anesthesiology.

### Increased Adherence to the ERAS Protocol

Implementation of an electronic notification significantly improved overall protocol adherence, and several preoperative markers of ERAS protocol adherence are shown in Table 2. The overall protocol adherence, use of oral gabapentin (600 mg), and oral celecoxib (400 mg) showed a statistically significant increase in use (Table 2). With respect to overall protocol adherence, 17% (14/85) of patients followed protocol in the pre-electronic notification group compared to 44% (44/99) of patients in the postelectronic notification group (*P*<.001). Among those in the pre-electronic notification group, 11% (11/85) received gabapentin, whereas in the postelectronic notification group, 43% (43/99) received gabapentin (*P*<.001). Lastly, 14 patients received celecoxib in the prenotification group, whereas 35 received it in the postnotification group (*P*=.006). There were no statistically significant differences in the use of scopolamine transdermal patch (*P*=.05), intraoperative ketamine (*P*=.36), and oral acetaminophen (*P*=.31) between the groups.

**Table 2:** Impact on each element of protocol.

Intervention	Pre-electronic notification, n (%)	Postelectronic notification, n (%)	*P* value
Protocol adherence	14 (17)	44 (44)	<.001
Gabapentin	11 (13)	43 (43)	<.001
Scopolamine	25 (29)	44 (44)	.05
Ketamine	3 (4)	7 (7)	.35
Celecoxib	14 (17)	35 (35)	.006
Acetaminophen	7 (8)	14 (14)	.31

### Secondary Outcomes

Next, we analyzed postoperative outcomes of patients undergoing breast surgery stratifying according to whether their anesthesia provider received an electronic notification for the ERAS protocol (Table 3). Secondary outcomes were intraoperative and postoperative morphine equivalent administered, PACU LOS, postoperative pain scores, and incidence of PONV. We did not show any statistically significant improvements in secondary patient outcomes.

**Table 3:** Secondary outcomes analysis.

Postoperative outcomes	Pre-electronic notification	Postelectronic notification	*P* value
Intraoperative morphine equivalent, median (IQR)	20.00 (10.00-30.00)	20.00 (10.00-30.00)	.55
Postoperative morphine equivalent, median (IQR)	6.11 (1.11-11.11)	6.11 (2.22-12.22)	.70
PACU^a^ LOS^b^, median (IQR)	118.00 (92.00-162.00)	154.00 (84.00-217.50)	.29
First pain score, median (IQR)	4.00 (2.00-6.00)	4.00 (2.00-6.00)	.39
Highest pain score, median (IQR)	5.00 (4.00-7.00)	5.00 (3.00-7.00)	.95
PONV^c^, n (%)	27 (32)	39 (39.4)	.36

^a^PACU: postanesthesia care unit.

^b^LOS: length of stay.

^c^PONV: postoperative nausea and vomiting.

The median postoperative morphine equivalent was 6.11 (IQR 1.11-11.11) for the prenotification group (85 patients) and 6.11 (IQR 2.22-12.22) for the postnotification group (99 patients), and there was no statistically significant difference in postoperative morphine equivalent between prenotification and postnotification groups (*P*=.70). The median intraoperative morphine equivalent was 20.00 (IQR 10.00-30.00) for both the prenotification and the postnotification groups of patients, and there was no statistically significant difference in intraoperative morphine equivalent between prenotification and postnotification groups (*P*=.55). LOS in the PACU was not statistically significant between the 2 groups, with the pre-electronic notification group having a median of 118.00 (IQR 92.00-162.00) minutes and postelectronic notification group having a median of 154.00 (IQR 84.00-217.50) minutes (*P*=.29). The median of the first recorded pain score was 4.00 (IQR 2.00-6.00) for both the pre-electronic notification group and postelectronic notification group (*P*=.31). The median highest recorded pain score was 5.00 (IQR 4.00-7.00) for the pre-electronic notification group and 5.00 (IQR 3.00-7.00) for the postelectronic notification group (*P*=.95). Lastly, the incidence of PONV was 27 (32%) among the pre-electronic notification group and 39 (39%) among the postelectronic notification group (*P*=.36).

## Discussion

### Principal Findings

This study exhibited statistically significant primary outcome measures estimating adherence to ERAS protocols determined by pre- and intraoperative use of medications but did not exhibit statistically significant secondary outcome measures. Recent studies have demonstrated benefits with the implementation of ERAS protocols in a variety of surgeries including cardiothoracic, gastrointestinal, and gynecologic surgery. Benefits of ERAS protocols included shorter LOS, more rapid return of bowel function, decreased postoperative pain, and increased patient satisfaction [2,3,9]. With respect to breast surgery, ERAS protocols have been shown to reduce the length of hospital stay, opioid use, and PONV without increasing the rates of complication [4,10-12].

Previous studies have also demonstrated the importance of decision support systems (DSS) in the clinical setting. The purpose of DSS is to aid clinicians in centralizing the increasing amounts of data for each patient alongside the increasing volume of medical research [13]. There are several types of CDSS often categorized based on the following characteristics: system function, model for advice, human interaction, and underlying decision-making process [14]. Our method of CDSS closely resembled the model for advice. This model, however, can be classified into 2 subcategories: passive and active. Passive DSS require the user to perform an action to receive advice. Active DSS, the model used in our study, involve the generation of alerts to providers as a means of decision support [14]. The latter has been shown to be efficacious in the perioperative phase. For instance, Kooij et al [15] demonstrated how electronic CDSS increased guidelines adherence for prescribing PONV prophylaxis. Our study sought to further investigate the benefits of CDSS in implementing ERAS protocols in patients undergoing major breast surgery.

Our analysis showed an improvement in multiple preoperative markers of the ERAS protocols suggesting improved guideline adherence. For instance, there was an increase in the use of gabapentin and celecoxib. This statistically significant difference from our pre- and postintervention groups supports our primary hypothesis that an electronic notification system will impact the frequency of which providers incorporate the breast surgery ERAS protocols. Other studies have shown similar results. One study examined the implementation of electronic alerts for improving adherence to foot exam screenings in type 2 diabetic patients in primary care clinics [16]. The researchers demonstrated that the use of an electronic clinical reminder to providers increased adherence and subsequently resulted in clinically significant outcomes. The aid of ERAS protocols has also been investigated in the perioperative setting where electronic DSS increased guideline adherence for the prescription of PONV prophylaxis and in the intraoperative setting where real-time electronic reminders improved compliance to institutional glucose management [14,17].

Although there was a statistically significant increase in the use of gabapentin and celecoxib, our analysis showed no statistically significant difference in the use of transdermal scopolamine, ketamine, or acetaminophen in the pre- and postintervention groups. This can be due to several reasons. For instance, with ketamine, there is limited evidence of its use in breast surgery [18]. In a randomized controlled trial evaluating for this effect, patients undergoing mastectomies were randomized to receive ketamine (0.15 mg/kg IV) before surgery or during closure. The researchers concluded that ketamine at the end of surgery was more effective in reducing morphine consumption as patient-controlled analgesia was lower during the first 2 hours in patients given ketamine at closure [18]. With the abundance of evidence that ketamine provides preoperative analgesia for multiple surgical procedures, it is probable that ketamine may be beneficial for breast surgery. However, a dearth of evidence specific to breast surgery may cause anesthesiology providers to preclude it from their practice [18].

With respect to acetaminophen, the lack of a statistically significant difference is likely due to changes in the preferred route of administration over the past few years. During the time of this study, intravenous acetaminophen was perceived as the gold standard for multimodal pain management at our institution. Consequently, only a minority of our patients, less than 15%, in both the pre- and postintervention groups, received oral acetaminophen as recommended per the ERAS protocols. However, recent studies have shown oral acetaminophen given preoperatively was equivalent to intravenous acetaminophen in controlling pain in the immediate postoperative phase [19]. Intravenous acetaminophen was also not found to be superior to oral acetaminophen in reducing time to ambulation, length of PACU stay, or PONV [19]. Consequently, intravenous acetaminophen was not included in our adherence calculation and only oral dosing was included as a factor for adherence. Given the clinical practice changes at our institution over recent years, if our study was done in the present, it is likely that the vast majority of our patients would have received oral acetaminophen, although the presence of a statistically significant difference between the pre- and postintervention groups would still be in question.

Some studies have reported that the adoption of clinical decision support tools could have a significant impact on the performance of providers [20]. However, it is erroneous to establish an equivalence between provider behaviors and patient outcomes. Research has shown it is imperative to assess the impact of clinical decision support on provider behavior and objective clinical end points [5]. Additionally, it is important to note DSS are only 1 component of increasing compliance and that increasing guideline adherence is multifactorial. This study shows that CDSS can be a meaningful component of a multifactorial protocol adherence strategy. Using traditional human and system factors to enforce clinical guidelines often results in suboptimal guideline adherence [21]. The goal of our electronic notification system was to augment the traditional systems in place that remind providers about existing protocols, in this case, an ERAS protocol for breast surgery. Our study also adds to the existing literature by analyzing secondary patient outcomes such as PACU LOS, morphine dosage, pain scores, and PONV stratifying according to whether the anesthesia provider received an electronic notification for the ERAS protocol. Interestingly, none of these variables showed statistical significance between the pre- and postnotification groups. This finding is consistent with the existing literature on perioperative clinical decision support. As explained by Nair et al [22], these are complex multifactorial perioperative outcomes, and CDSS may represent merely 1 piece of a multifaceted quality improvement strategy.

### Limitations

The findings of this study should be interpreted in the context of the following limitations. Data are limited on variables such as the length of surgery, which could plausibly introduce bias. We also had to exclude 12 patients in the prenotification group that had minor breast surgery (ie, breast biopsy and lumpectomies). These 12 patients were also excluded from the secondary analysis, as well as excluding other 3 patients from the preintervention group and 1 patient from the postintervention group with missing data. Nonetheless, secondary postoperative patient outcomes were not found to be significant. Adherence to protocol was defined as a 20% increase in protocol use. Unfortunately, there is no defined standard to quantify adherence, and there is widespread variation of this metric across literature, but for the purpose of our study, we wanted to show an incremental increase in usage, knowing that gauging adherence is a multimodal approach. Furthermore, these findings are from data collected in a single large academic medical center and may not be representative of a larger cohort. Future studies, ideally multicenter, are needed to establish a more robust relationship between electronic notification systems and provider adherence.

### Conclusions

This study exhibited statistically significant primary outcome measures measuring adherence to ERAS protocols determined by pre- and intraoperative use of medications but did not exhibit statistically significant secondary outcome measures including intraoperative and postoperative narcotic administered, PACU LOS, first pain score, highest pain scores postoperatively, and incidence of PONV. Although existing studies have identified the benefits of ERAS protocols, several barriers are present, which prevent the practice of these guidelines. Our study demonstrates that 1 technique to overcome this is by using automated notifications for anesthesia care providers. This paper contributes to the existing literature by examining how electronic notifications could increase adherence to ERAS protocol use and shows how one can build a relatively simple CDSS using our methodology to do so. In the future, we hope to see this model repeated by anesthesiologists across the country with various alterations to examine other areas in which patients may benefit from decision support systems.

## Appendix

Multimedia Appendix 1 ERAS protocol used for breast surgery sent to surgeons and anesthesiologists.

## Abbreviations

ASA: American Society of Anesthesiology
CDSS: clinical decision support system
DSS: decision support system
ERAS: enhanced recovery after surgery
LOS: length of stay
PACU: postanesthesia care unit
PONV: postoperative nausea and vomiting
