# Supplementary material for: Efficacy of Electronic Reminders in Increasing the Enhanced Recovery After Surgery Protocol Use During Major Breast Surgery: Prospective Cohort Study
Source: JMIR Perioper Med. 2023 Nov 3;6:e44139. doi: 10.2196/44139 (PMC10656665; doi:10.2196/44139)
Supplement: Multimedia Appendix 1 [file periop_v6i1e44139_app1.pdf]

Medstar Georgetown University Hospital

Breast Surgery/Anesthesia Enhanced Recovery After Surgery (ERAS) Pathway

Anesthesia Equipment

- BIS Monitor

Preoperative Clinic

- Surgical Clinic
  - Explain surgical approach and enhanced recovery criteria and expectation
  - Explain npo instructions
  - Schedule Anesthesia Preop Clinic visit same day as surgeon office visit if indicated
  - Smoking cessation counselling

- Anesthesia Clinic

Optimize Hgb
  - Draw labs (CBC, BMP, coags)
  - Reinforce ERAS
  - Clear liquid/high carbohydrate drink up to 2 hours prior to surgery (20 oz gatorade)
    - Phone call week leading up to surgery to ensure patient's questions answered

Preoperative Holding

- Reinforce NPO: can drink up to 2 hours before surgery
- Gatorade given prior to nuclear medicine appt
  - Will be available in sds holding
- Anesthesia team to order preop meds if indicated
- **GABAPENTIN 600 mg PO**
- **CELEBREX 400mg PO --Contraindicated if SULFA allergy, CrCl<30 mL/min**
- **AM Admit/ 23hr Obs -ACETAMINOPHEN 1000 mg PO**
- **Scopolamine patch** if GA planned

Intraoperative

Antibiotics
-**Surgeon preference**

▸ For local/MAC case:
- **Midazolam PRN**
- **Propofol gtt**
- **Consider Ketamine 20mg IV +/- gtt at 0.2mg/kg/hr**

▸ For general case:
- **Consider TIVA for cases <2 hours or for patients with severe PONV**
  - **BIS Monitor for all GA/TIVA**
- Ketamine 20mg IV +/- gtt at 0.2mg/kg/hr
- Decadron 8mg IV at induction
- Ondansetron 4mg IV prior to extubation
- Acetaminophen 1000mg IV (If not given preop)

DVT prophylaxis--SCDs and early ambulation post op

Postoperative

▸ **Day of Surgery**

SDS
- **Fentanyl PRN**
- **Zofran/ Phenergan PRN**

AM ADMIT
- **Fentanyl PRN**
- **Zofran/Phenergan PRN**
- **ACETAMINOPHEN** 1000 mg po q8h scheduled
- **GABAPENTIN** 300 mg po q8h
